# Inflammatory pseudotumor of the spleen concomitant with renal cell carcinoma: case report

**DOI:** 10.1590/S1516-31802004000500008

**Published:** 2004-09-01

**Authors:** Sérgio Ossamu Ioshii, Danielle Giacometti Sakamoto, Tiago Noguchi Machuca, Ryuichi Yatani

**Keywords:** Spleen, Plasma cell granuloma, Renal cell carcinoma, Immunohistochemistry, Neoplasms, Baço, Pseudotumor inflamatório, Carcinoma de células renais, Imunohistoquímica, Neoplasias

## Abstract

**CONTEXT:**

Inflammatory pseudotumor is a rare benign lesion that can occur at a wide variety of primary sites. It is usually worrisome for the patient and the medical staff, since it cannot be clinically or radiologically distinguished from malignant entities.

**CASE REPORT:**

We report on a case of splenic inflammatory pseudotumor presenting with concomitant renal cell carcinoma. Despite the alarming macroscopic appearance of pseudotumors, their microscopic features usually confirm the inflamma-tory nature of such lesions. Evidence regarding the etiology of pseudotumors is still lacking, but hypotheses have been created.

## RESUMO

**CONTEXTO:**

Pseudotumor inflamatório é uma lesão benigna rara que pode se desenvolver em uma grande variedade de órgãos. Sua grande importância na prática médica reside na enorme dificuldade de distingui-lo tanto clinicamente quanto radiologicamente de condições malignas.

**RELATO DE CASO:**

Os autores relatam um caso de pseudotumor envolvendo o baço e apresentandose concomitantemente a um carcinoma renal de células claras. Apesar da semelhança macroscópica a tumores malignos, as características inflamatórias do pseudotumor são facilmente evidenciadas à microscopia. Apesar de hipóteses terem sido formuladas, os mecanismos patogenéticos relacionados ao pseudotumor ainda não estão bem estabelecidos.

## INTRODUCTION

Inflammatory pseudotumors are rare lesions that can occur in virtually any organ. Their greatest significance is in relation to the inability to distinguish splenic inflammatory pseudotumors from malignant conditions, based solely on their clinical and radiological appearance. Splenic inflammatory pseudotumors are extremely rare, with only approximately 60 cases reported in the literature. Histologically, it is characterized by nonspecific and reparative changes resembling granulation tissue.^1^ Cases presenting with a concomitant neoplasm raise the suspicion of metastatic spread and are difficult to manage in the clinical setting, since accurate preoperative staging is, in most cases, impossible. We report on a case of splenic inflammatory pseudotumor presenting with concomitant renal cell carcinoma.

## CASE REPORT

**Figure 1:**
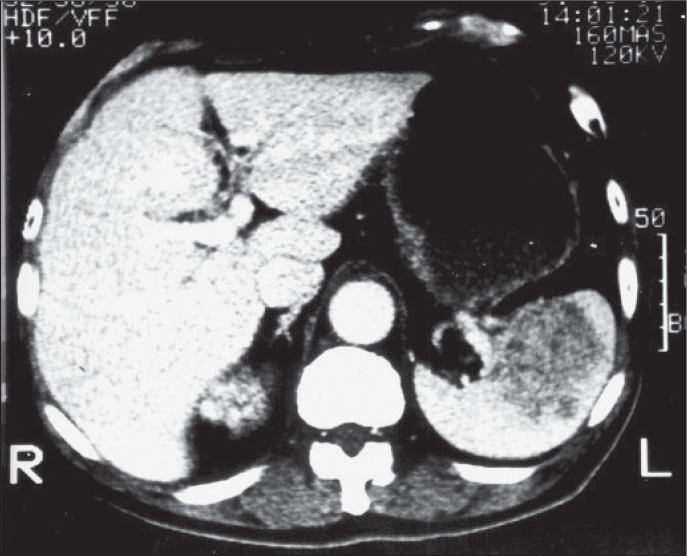
Computerized tomography displaying a lobulated splenic mass suggestive of a metastatic malignancy in a 66-year-old man.

A 66-year-old man presented with complaints of a pelvic mass. Computerized tomography revealed a nodular tumor at the lower pole of the left kidney and an irregularly lobulated low-density splenic mass suggestive of splenic metastasis (Figure 1). No lymph node enlargement was observed. Angiographic examination of the left kidney showed a pattern highly suggestive of carcinoma. The patient underwent nephrectomy and, since no other site of systemic involvement was observed on computerized tomography scan, the spleen was also removed.

### Macroscopic findings

The left kidney was deformed by an 8 x 7 x 6 cm soft white nodular mass at its lower pole. The tumor did not present renal capsule invasion, but renal vein involvement was present. A small metastasis was observed in the cortex, near the primary tumor. No hilar lymph nodes were positive for malignancy.

The spleen weighed 135 g and was altered by an irregularly lobulated white mass of hard consistency, measuring 10 cm in diameter. There were brown and yellow areas suggestive of old hemorrhage. No signs of thrombosis were observed in the hilar blood vessels.

### Histological findings

Formalin-fixed paraffin-embedded tissue sections were obtained from both the renal and the splenic tumors, and were stained with hematoxylin-eosin. Histo-chemical analysis for reticulin fibers was also performed.

Microscopic evaluation of the renal tumor showed cells with clear cytoplasm and distinct cell membranes. The nuclei were slightly irregular and nucleoli were prominent, leading to a diagnosis of grade 2 clear cell renal carcinoma (Fuhrman grading system).

**Figure 2:**
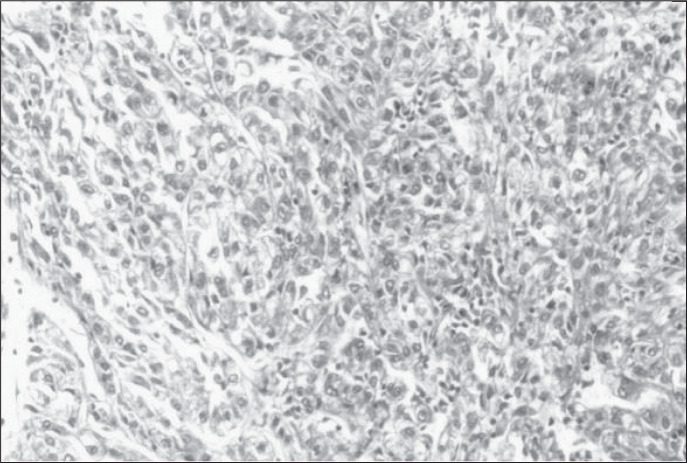
Photomicrography showing fibroblasts and lymphocytes of benign appearance, thus confirming the inflammatory nature of the tumor (hematoxylin-eosin, 200 x).

Histological examination of the splenic mass showed irregular dense collagen bundles with zones of fibroblastic proliferation in association with areas with lymphocytes, plasma cells and deposits of hemosiderin (Figure 2). Some areas showed concentric deposition of collagen in an “onion-skin” pattern. Reticulin staining showed islands of atrophic splenic tissue within the inflammatory lesion.

The histological examination was suggestive of an inflammatory pseudotumor, but in order to rule out a possible meta-static focus from the renal lesion, immunohistochemical analysis was performed. The avidin-biotin-peroxidase method was employed and the results confirmed our suspicion (Table 1).

**Table 1:** Immunohistochemical analysis of the renal and splenic lesions of a 66-year-old man

Antibody	source/dilution	renal lesion	splenic lesion
CKAE1/AE3	Dako/1:400	positive	negative
Vimentin	Dako/1:1000	positive, focal	positive
Actin	Dako/1:3000	negative	positive
Desmin	Dako/1:200	negative	positive
CD34	Dako/1:600	negative	positive

*CKAE1/AE3 = high and low-molecular weight cytokeratin.*

## DISCUSSION

Splenic masses are most suggestive of lesions such as lymphoma, hematoma, abscess or infarct. Splenic inflammatory pseudotumors are rare, with only approximately sixty cases reported in the indexed world literature. The clinical presentation of splenic inflammatory pseudotumors is usually related to nonspecific signs and symptoms, or they may be detected incidentally during diagnostic workup for other diseases or during periodic clinical evaluation. The most frequent symptoms are abdominal pain, fatigue, weight loss, nausea, dyspepsia and night sweating.^2^ Among the signs, fever and leukocytosis are often present.^2^

Imaging studies of splenic inflammatory pseudotumors are as nonspecific as their clinical features. Plain radiography may show splenomegaly. Echography reveals that the tumor is usually hypoechoic and well demarcated. Computerized tomography with intravenous contrast medium may show a central stellate area corresponding to a fibrous plaque. This appearance has been reported as suggestive of splenic inflammatory pseudotumor.^3^ However, it is not possible to clearly detect splenic inflammatory pseudotumors solely on the basis of radiological data.

The treatment of splenic inflammatory pseudotumors consists of splenectomy.^1^ The published long-term follow-ups were uneventful and, to the best of our knowledge, no recurrence has been described in the literature. There have not been any cases of splenic inflammatory pseudotumors presenting or evolving with metastasis.

Because of their benign nature and favorable clinical course, the greatest dilemma regarding splenic inflammatory pseudotumors is in relation to whether splenic malignancies can be ruled out, thereby establishing pseudotumor as the correct diagnosis. Because of the nonspecific clinical presentation and unremarkable radiological appearance of pseudotumors, such distinction can only be established on the basis of histological analysis. Even in the absence of characteristic features such as nodal and bone marrow involvement, the most common preoperative diagnosis in splenic inflammatory pseudotumor cases is splenic lymphoma.^1^ In addition to lymphomas, the lesions frequently included in the differential diagnosis for splenic inflammatory pseudotumors are: lymphoreticular malignancies other than lymphomas (e.g. solitary plasmacytoma), hemangioma, tumoral proliferation of follicular dendritic reticulum cells, splenic hamartoma, reactive lymphoid hyperplasia and malignant fibrous histiocytoma.

In spite of the good demarcation presented by the tumor, it is not possible to rule out a malignant condition through macroscopic examination. Such a distinction can usually be achieved histologically. Upon microscopic examination, the tumor appears polymorphous, with spindle cells, a variable quantity of fibrocollagenous stroma and a granulomatous inflammatory component. There is predominance of lymphocytes (mostly T cells), plasma cells, histiocytes and, to a lesser extent, neutrophils and eosinophils.^1,2^ The features of the lymphocytes emphasize the benign nature of the tumor, with small, cytologically mature cells, absence of nuclear atypia and low mitotic activity. Centrally located areas of coagulative necrosis and areas of hemorrhage are often found.^2^

The etiology of splenic inflammatory pseudotumors remains unknown. Evidence favoring a possible infectious stimulus is still lacking. Except for sporadic reports, special stains and microbiological culturing have failed to detect an etiological agent.^4^ Their concomitant presentation with idiopathic thrombocytopenic purpura and the architectural similarities with Riedel's thyroiditis and idiopathic retroperitoneal fibrosis may point towards autoimmune pathogenesis.^5^ Nevertheless, further studies are necessary in order to support this hypothesis.

In summary, splenic inflammatory pseudotumors are rare benign lesions that should be considered when evaluating patients with splenic masses. The correct diagnosis is only made on the basis of microscopic findings and splenectomy is curative. The pathogenetic mechanisms involved in splenic inflammatory pseudotumors are not clear, but infectious and autoimmune hypotheses have been put forward.
